# circ_0003204 regulates the osteogenic differentiation of human adipose-derived stem cells via miR-370-3p/HDAC4 axis

**DOI:** 10.1038/s41368-022-00184-2

**Published:** 2022-06-21

**Authors:** Liyuan Yu, Kai Xia, Jing Zhou, Zhiai Hu, Xing Yin, Chenchen Zhou, Shujuan Zou, Jun Liu

**Affiliations:** 1 State Key Laboratory of Oral Diseases & National Clinical Research Center for Oral Diseases & Department of Orthodontics, West China Hospital of Stomatology, Sichuan University, Chengdu, China; 2 Department of Stomatology, Kunming Yan’an hospital, Kunming, China; 3 State Key Laboratory of Oral Diseases & National Clinical Research Center for Oral Diseases & Department of Pediatric Dentistry, West China Hospital of Stomatology, Sichuan University, Chengdu, China

**Keywords:** RNA, Molecular medicine

## Abstract

Human adipose-derived stem cells (hASCs) are a promising cell type for bone tissue regeneration. Circular RNAs (circRNAs) have been shown to play a critical role in regulating various cell differentiation and involve in mesenchymal stem cell osteogenesis. However, how circRNAs regulate hASCs in osteogenesis is still unclear. Herein, we found circ_0003204 was significantly downregulated during osteogenic differentiation of hASCs. Knockdown of circ_0003204 by siRNA or overexpression by lentivirus confirmed circ_0003204 could negatively regulate the osteogenic differentiation of hASCs. We performed dual-luciferase reporting assay and rescue experiments to verify circ_0003204 regulated osteogenic differentiation via sponging miR-370-3p. We predicted and confirmed that miR-370-3p had targets in the 3′-UTR of HDAC4 mRNA. The following rescue experiments indicated that circ_0003204 regulated the osteogenic differentiation of hASCs via miR-370-3p/HDAC4 axis. Subsequent in vivo experiments showed the silencing of circ_0003204 increased the bone formation and promoted the expression of osteogenic-related proteins in a mouse bone defect model, while overexpression of circ_0003204 inhibited bone defect repair. Our findings indicated that circ_0003204 might be a promising target to promote the efficacy of hASCs in repairing bone defects.

## Introduction

Bone defects can result from congenital malformation, trauma, inflammation, or surgical resection, which may cause severe functional, esthetic, and psychological problems. The osseous repair of bone defects is still challenging in the field of orthopedics and maxillofacial surgery. The usage of autogenous bone graft is associated with limited availability, donor-site pain and morbidity. Most of the alternative bone grafts, irrespective of allogenic, xenogenic, and synthetic ones, lack osteogenic elements, thus cannot be used alone to repair bone defects.^1^ One promising approach is to adopt human adipose-derived stem cells (hASCs) to confer bone grafts potent osteogenic elements.

With the development of stem cell therapy, mesenchymal stem cells (MSCs) are regarded as a tool for bone regeneration therapies. hASCs, a desirable cell source of MSCs, possess the great advantages of minimally invasive procedure, high production, and multiple differentiation.^2^ Therefore, they have great potential for application in cell‐based therapies. Efforts have been invested in elucidating the molecular mechanisms and signaling pathways related to hASC osteogenesis. It has been shown that lncRNA-PCAT1 increased the osteogenic differentiation of hASCs *via* targeting miR-145-5p.^3^ CircPOMT1 and circMCM3AP were reported to target miR-6881-3p and inhibit hASC osteogenesis.^4^ These data suggested that noncoding RNAs plays major roles in the osteogenic differentiation of hASCs.

Circular RNAs (circRNAs) are a distinct type of nonlinear, noncoding RNAs, whose 3’- and 5’-ends are covalently bonded to form a continuous closed-loop, thus resisting RNA exonucleases digestion.^5^ In the past few years, circRNAs are considered as a crucial regulatory factor in cell differentiation and participate in regulating MSC osteogenesis. Previous studies found that circCDK8 influences osteogenesis and apoptosis of periodontal ligament stem cells (PDLSCs) through inducing endoplasmic reticulum stress/autophagy under hypoxia.^6^ Another study confirmed that circ_0074834 enhances the osteo-adipogenic differentiation of bone mesenchymal stem cells (BMSCs) through serving as a miRNA sponge for miR-942-5p.^7^ Our preliminary study found that circRFWD2 and circINO80 play vital regulated roles in NELL-1-induced hASC osteogenesis.^8^ Among the various regulatory mechanisms of circRNAs, the theory of competitive endogenous RNA (ceRNA) has attracted extensive attention. According to this theory, circRNAs efficiently bind to miRNAs by base complementation, thus block the inhibitory effects of miRNAs on their target genes, and indirectly promote their expression. For example, circRNA124534 can sponge miR-496 and consequently regulate β-catenin during human dental pulp stromal cell (hDPSC) osteogenesis.^9^ circRNA SIPA1L1 was reported to promote DPSC osteogenesis through the miR-617/Smad3 axis.^10^ Moreover, hsa_circRNA_33287 sponges miR-214-3p and regulates Runx3 expression during maxillary sinus membrane stem cell osteogenesis.^11^ Numerous researches have revealed that circRNAs regulate ASC osteogenesis through sponging miRNAs. Zhang et al. confirmed that circRNA-vgll3 increased hASC osteogenesis by regulating miRNA-dependent integrin α5 expression.^12^ Another study verified that circPOMT1 and circMCM3AP inhibit hASC osteogenesis through miR-6881-3p.^4^ These findings suggest that circRNAs are most likely to play a role as intermediate factors in ASC osteogenesis via sponging miRNAs.

To investigate the effect of circRNA-transfected hASCs on bone regeneration, scaffolds with good biocompatibility and optimal structural properties are indispensable. Emerging from the rapid advances in material science research, hydrogel-based biomaterials as cell culture scaffolds have a broad application potential in tissue engineering.^13^ Methacryloylated gelatin (GelMA) is a double bond modified gelatin, which can be cross-linked under ultraviolet (UV) or visible light with a suitable photoinitiator, such as Irgacure or lithium phenyl-2,4,6-trimethylbenzoylphosphinate (LAP).^14^ As a cell culture matrix that mimics native extracellular matrix, GelMA is widely used in the fields of cell culture and tissue engineering with good biocompatibility for cell adhesion, diffusion, and proliferation. It is reported that GelMA combined with hDPSCs and human umbilical vein endothelial cells can regenerate human dental pulp tissue.^15^ In addition, Chen et al. reported the repair of osteochondral defects with 3D-printed GelMA-exosome scaffolds.^16^ Therefore, in this study, transfected cells were encapsulated in GelMA to evaluate the in vivo effect of circRNA on bone regeneration.

Preliminary research has identified that circRNAs and miRNAs were differentially expressed during the osteogenic differentiation of hASCs and predicted the potential circRNA-miRNA ceRNA mechanism.^4,17^ According to the preliminary results, we focused on circ_0003204 which was downregulated in hASC osteogenesis. Its potential target miR-370-3p was also proved to induce osteogenic differentiation of hASCs. In this study, we attempted to examine the regulatory mechanism of circ_0003204 in hASC osteogenesis by both in vivo and in vitro experiments.

## Results

### circ_0003204 negatively regulated hASC osteogenesis

Previous studies have confirmed the osteogenic capacity of hASCs as indicated by alkaline phosphatase (ALP) staining, alizarin red s (ARS) staining, and RT-qPCR assay.^4^ Current results from immunofluorescence staining (IF) further demonstrated that hASC osteogenic induction was also accompanied with upregulated expressions of RUNX2 and COL1A1 (Supplementary Fig. S1a, b). In our preliminary research, we have identified differentially expressed circRNAs during the osteogenesis of hASCs and performed bioinformatic analysis.^4^ circ_0003204 was downregulated during the osteogenic differentiation of hASCs and might play a crucial regulatory role. RT-qPCR data confirmed that the expression of circ_0003204 was significantly decreased in the group of osteogenic induction 7 days (referred to as “Osteo”) in comparison with the control group (Supplementary Fig. S2a). To further verify the location of circ_0003204 in hASCs, we performed the separation of cytoplasmic and nuclear fractions. The RNA expression levels in cytoplasmic and nuclear fractions indicated circ_0003204 was mainly detected in the cytoplasm of hASCs (Supplementary Fig. S2b).

The overexpression lentivirus of circRNA_0003204 (Lv-circ), circRNA_0003204 siRNA (si-circ), and negative control (Lv-NC and si-NC) were separately transfected into hASCs to examine the function of circ_0003204 in hASC osteogenesis. After 24 h transfection with Cy3‐labeled siRNA and 72 h transfection with ZsGreen lentivirus, fluorescence images showed successful transfection (Supplementary Fig. S2c). The results of RT-qPCR analysis confirmed that circ_0003204 expression was reduced in si-circ in comparison with si-NC (Supplementary Fig. S2a). Subsequently, compared with Lv-NC, the circ_0003204 expression levels were obviously increased in Lv-circ.

**Fig. 1:**
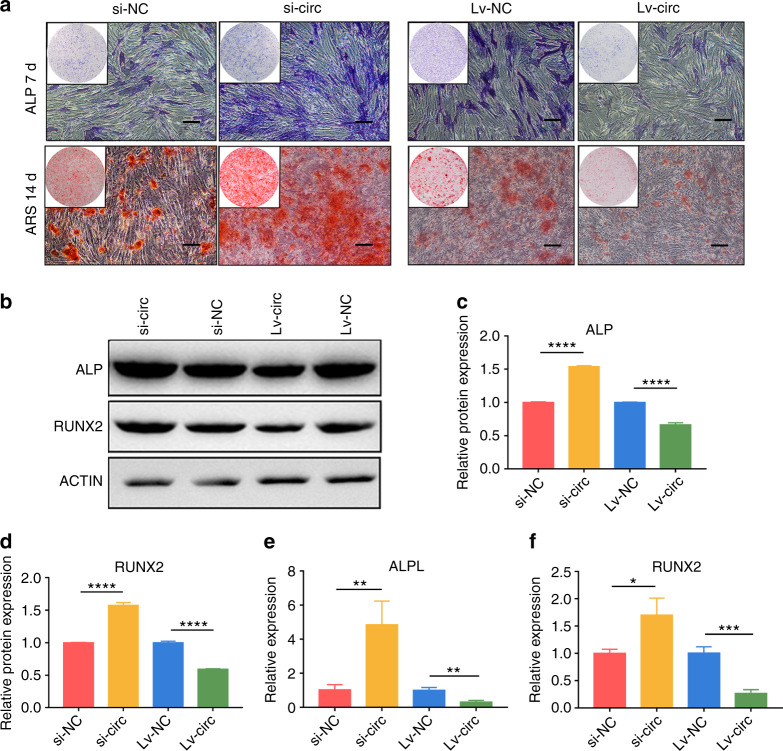
circ_0003204 negatively regulated hASC osteogenesis. **a** Images of ALP and ARS stainings after transfection (scale bar = 200 μm). **b**–**d** Western blot after 7-day osteogenic induction of hASCs detected the protein expressions and the results were quantitated. **e**, **f** The expressions of ALPL and RUNX2 after 7-day osteogenic induction of hASCs were evaluated by RT-qPCR. (*P < 0.05, **P < 0.01, ***P < 0.001, ****P < 0.000 1)

**Fig. 2:**
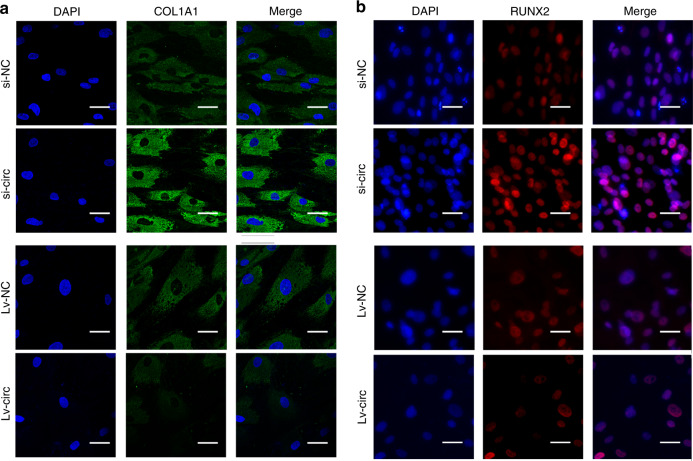
Images of immunofluorescence staining after 7-day osteogenic induction of hASCs. **a** COL1A1was stained in green and nuclei were stained in blue (scale bar = 40 μm). **b** RUNX2 was stained in red and nuclei were stained in blue (scale bar = 40 μm)

After a successful transfection with lentivirus or siRNA, hASCs were incubated in osteogenic induction medium. The ALP and ARS stainings significantly reduced in the group of Lv-circ when compared to Lv-NC, while it was enhanced in the group of si-circ in comparison with si-NC (Fig. 1a). Western blot showed that the protein expression levels of ALP and RUNX2 were also significantly upregulated in the group of si-circ and markedly downregulated in the group of Lv-circ (Fig. 1b–d). Consistent results were also obtained in RT-qPCR analyses (Fig. 1e, f) of the ALPL and RUNX2. Furthermore, IF staining revealed both RUNX2 and COL1A1 were downregulated in circ_0003204 overexpression, while upregulated in circ_0003204 knockdown (Fig. 2a, b). Taken together, circ_0003204 negatively regulates the osteogenic differentiation of hASCs.

### circ_0003204 inhibited hASC osteogenesis via sponging miR-370-3p

**Fig. 3:**
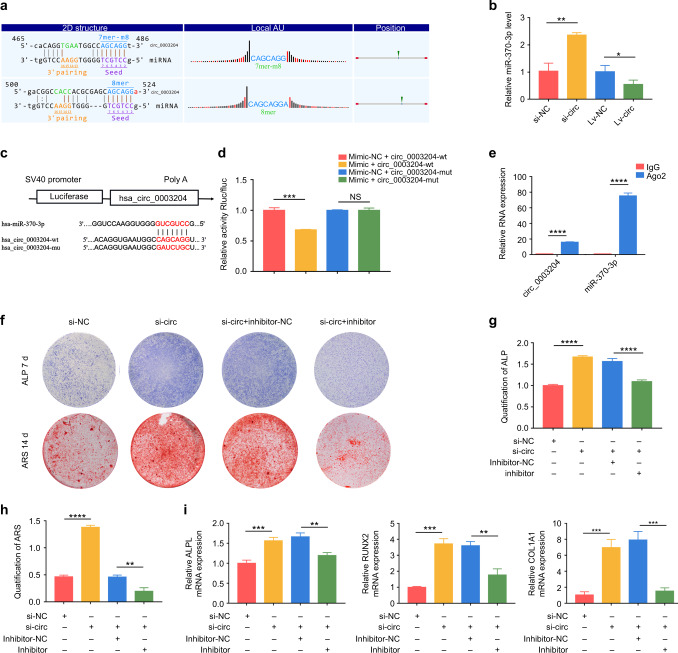
circ_0003204 inhibited hASC osteogenesis through sponging miR-370-3p. **a** Bioinformatic analysis predicted the target relationship. **b** RT-qPCR evaluated the expression of miR-370-3p. **c** The putative binding sequences. **d** Luciferase reporter genes were co-transfected with miR‐370‐3p mimic/mimic-NC and mut-type/wild-type circ_0003204 into cells, and the luciferase activities were measured. **e** RT-PCR evaluated the expression of circ_0003204 and miR-370-3p. **f** Images of ALP and ARS stainings after transfection. **g**, **h** ALP and ARS activity analyses were measured. **i** RT-qPCR evaluated the mRNA expressions after 7-day osteogenic induction of hASCs (*P < 0.05, **P < 0.01, ***P < 0.001, ****P < 0.000 1)

We used Starbase v2.0, TargetScan 7.2, and miRanda to find potential targets of circ_0003204 to explore whether circ_0003204 functions as a ceRNA. We identified that miR-370-3p contained two highly matched binding sites in circ_0003204 (Fig. 3a). The results of RT-qPCR analysis showed a decrease of miR-370-3p expression after circ_0003204 overexpression, while an increase after circ_0003204 down-expression (Fig. 3b). The results showed that circ_0003204 expression was negatively correlated with miR-370-3p during the osteogenic differentiation of hASCs. A dual-luciferase reporting assay was then applied to verify the direct target relationship. The hsa-circ_0003204-wt (wild-type) and hsa-circ_0003204-mut (mutations) in the predicted binding sequence were transfected into the dual-luciferase reporting plasmids (PSI-CHECK2) (Fig. 3c). Likewise, PSI-CHECK2 were co-transfected with miR‐370‐3p mimic and mimic negative control (referred to as “mimic” and “mimic-NC”, respectively). miR-370-3p significantly reduced the luciferase activity in hsa_circ_0003204-wt transfected cells, compared with the mimic-NC (Fig. 3d). In contrast, the luciferase activity had no significant difference in hsa_circ_0003204-mut transfected cells. Furthermore, RNA immunoprecipitation (RIP) assay showed that circ_0003204 and miR-370-3p were in the same RNA-induced silencing complex (RISC). RT-PCR results showed that circ_0003204 and miR-370-3p were enriched in Ago2 immunoprecipitation complex (Fig. 3e).

Since circ_0003204 functions as a ceRNA for miR-370-3p, si-circ, and miR-370-3p inhibitor (referred to as “inhibitor”) were co-transfected into hASCs. Si-NC and inhibitor negative control (referred to as “inhibitor-NC”) were conducted as controls, respectively. Co-transfected hASCs were conducted ALP, ARS stainings, and relative activity analyses after osteogenic induction. As shown by the increased ALP and ARS stainings and relative activity analyses (Fig. 3f–h), knockdown of circ_0003204 markedly promoted the osteogenic differentiation of hASCs, which could be markedly attenuated by silencing miR-370-3p. In addition, miR-370-3p inhibitor was observed to reverse the upregulation of the osteogenic genes (ALPL, RUNX2, and COL1A1), which were promoted by si-circ (Fig. 3i). These data indicated that circ_0003204 regulated osteogenic differentiation by sponging miR-370-3p.

### miR-370-3p regulated hASC osteogenesis by targeting HDAC4

**Fig. 4:**
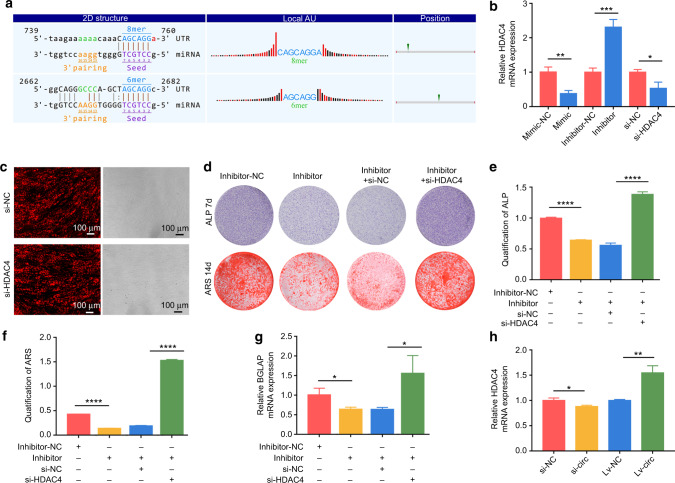
miR-370-3p regulated hASC osteogenesis by targeting HDAC4. **a** Bioinformatic analysis predicted the target relationship. **b** The expressions of HDAC4 were evaluated by RT-qPCR. **c** circ_0003204 siRNAs carried Cy3 reporter (red) and transfection efficiency was confirmed by fluorescence microscopy (scale bar = 100 μm). **d** Images of ALP and ARS stainings after transfection. **e**, **f** ALP and ARS activity analyses were detected. **g** RT-qPCR evaluated the mRNA expression levels of BGLAP after 7-day osteogenic induction of hASCs. **h** The expression of HDAC4 was regulated by circ_0003204. (*P < 0.05, **P < 0.01, ***P < 0.001, ****P < 0.000 1)

Our preliminary research confirmed the effects of miR-370-3p on mediating hASC osteogenesis.^17^ Other scholars found that miR-370-3p regulated hBMSC osteogenesis through WNT2B.^18^ In this study, we used TargetScan 7.2 and found that miR-370-3p had targets in the 3′-UTR of HDAC4 mRNA (Fig. 4a). Then, RT-qPCR results verified the miR-370-3p expression was negatively correlated with HDAC4 (Fig. 4b).

After 24 h of transfection with Cy3‐labeled siRNA HDAC4 (si-HDAC4) or negative control (si-NC), fluorescence images were obtained, indicating successful transfection (Fig. 4c). The RT-qPCR analysis determined HDAC4 expression was decreased in si-HDAC4 compared with si-NC (Fig. 4b). To further confirm the target relationship in hASC osteogenesis, we co-transfected miR-370-3p inhibitor and si-HDAC4 into hASCs. Si-NC and inhibitor-NC were conducted as respective controls. ALP and ARS stainings and relative activity analyses were conducted after the osteogenic induction of co-transfected hASCs. As shown by the decreased ALP and ARS stainings and relative activity analyses (Fig. 4d–f), knockdown of miR-370-3p markedly inhibited the osteogenic differentiation of hASCs, which was, whereas, markedly attenuated by silencing HDAC4. We also observed that the knockdown of HDAC4 reversed the down-regulation of BGLAP expression level, which was inhibited by the miR-370-3p inhibitor (Fig. 4g). Likewise, HDAC4 expression was downregulated in si-circ compared with si-NC, while it was upregulated in circ_0003204 overexpression (Fig. 4h). These data indicated that circ_0003204 regulated hASC osteogenic differentiation via the miR-370-3p/HDAC4 axis.

### HDAC4 inhibited hASC osteogenesis

**Fig. 5:**
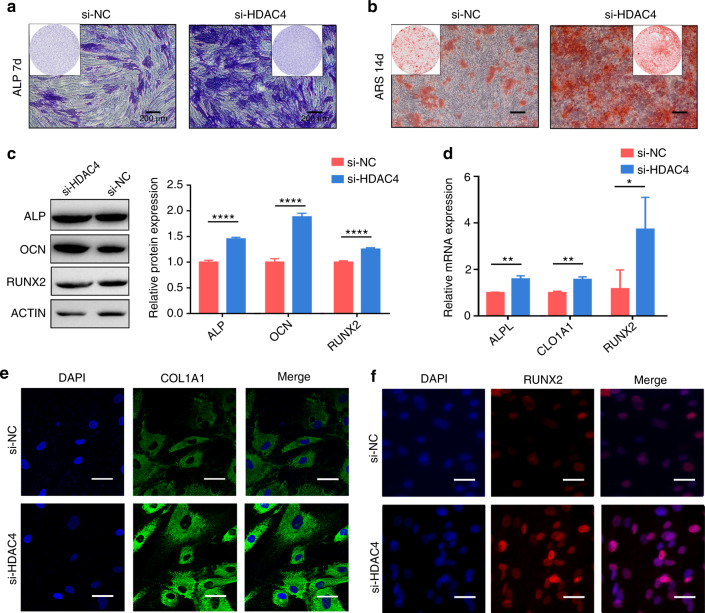
HDAC4 inhibited hASC osteogenesis. **a**, **b** Images of ALP and ARS stainings after transfection (scale bar = 200 μm). **c** Western blot evaluated the protein expressions after 7-day osteogenic induction of hASCs and histograms show quantification of the band intensities. **d** RT-qPCR evaluated the mRNA expressions after 7-day osteogenic induction of hASCs. **e**, **f** Immunofluorescence (IF) staining of COL1A1 (green) and RUNX2 (red) was performed after 7-day osteogenic differentiation (scale bar = 40 μm). The 4’,6-diamidino-2-phenylindole (DAPI) stained nuclei (blue) (*P < 0.05, **P < 0.01, ****P < 0.000 1)

The cells were grown in osteogenic induction medium after transfection. The ALP staining showed obviously enhanced after a 7-day induction in the si-HDAC4 group compared to the si-NC group (Fig. 5a). ARS staining after a 14-day induction revealed mineralized nodules formation was enhanced in the si-HDAC4 group (Fig. 5b). In addition, western blot results showed that ALP, OCN, and RUNX2 expressions were substantially promoted when HDAC4 was knocked down (Fig. 5c). The subsequent RT-qPCR analyses showed the expressions of ALPL, COL1A1, and RUNX2 genes (Fig. 5d) were promoted in HDAC4 knockdown. Furthermore, consistent results were also obtained in IF staining (Fig. 5e, f) of the COL1A1 and RUNX2. Taken together, HDAC4 inhibited the osteogenic differentiation of hASCs.

### circ_0003204 regulated hASC osteogenic differentiation in vivo

**Fig. 6:**
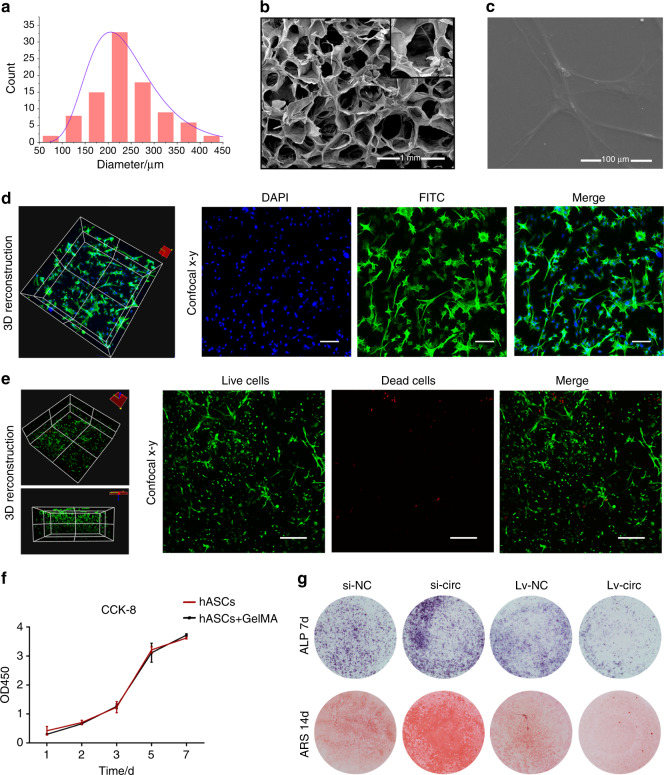
Characterization of GelMA and coculture of hASCs in GelMA. **a** The diameter distribution of GelMA. **b**, **c** The SEM image exhibited the three-dimensional structure of GelMA and the morphology of hASCs on the surface of GelMA. **d** Confocal microscopy was used to observe the morphology of hASCs encapsulated in the GelMA (scale bar = 100 μm). **e** Images of Calcein-AM/PI staining, where calcein-AM indicated live cells with green fluorescence and PI indicated dead cells with red fluorescence (scale bar = 200 μm). **f** Proliferation ability of hASCs encapsulated in GelMA scaffold was measured using CCK-8. **g** Images of ALP and ARS stainings after transfection

The scanning electron microscope (SEM) images exhibited that the GelMA has a three-dimensional porous and interconnected structure with an average pore diameter ranging from 180 to 230 μm (Fig. 6a, b). SEM analysis (Fig. 6c) showed that hASCs exhibited an elongated morphology and adhered tightly to the surface of GelMA and confocal microscopy (Fig. 6d) displayed a high cell density and actin filament spreading, indicating that the GelMA was favorable for the spreading of hASCs. Calcein-AM/PI staining was performed to detect the viability of hASCs that were encapsulated in GelMA after 3 days of culture. The images (Fig. 6e) indicated that cells remained alive and active. The proliferation of hASCs encapsulated in GelMA was measured using CCK-8. The results indicated that cells in each group proliferated over time, while there was no significant difference among the different groups at all time points (Fig. 6f). The transfected hASCs including Lv-circ, Lv -NC, si-NC, and si-circ were encapsulated in GelMA scaffolds and then cultured in osteogenic medium. ALP and ARS stainings (Fig. 6g) were significantly enhanced in the si-circ group, while there was no obvious staining in the Lv-circ group. The results indicated that circ_0003204 inhibited the osteogenic differentiation of hASCs that were encapsulated in GelMA.

**Fig. 7:**
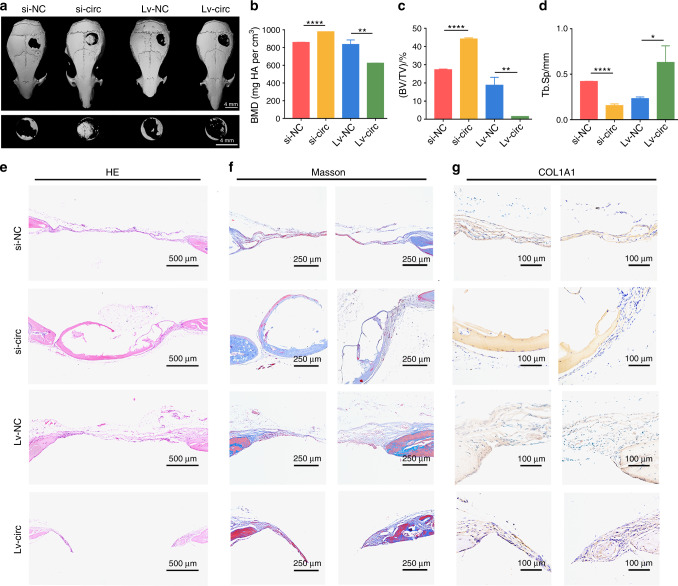
circ_0003204 regulated hASC osteogenic differentiation in vivo. **a** Three-dimensional (3D) reconstructed images of the defect sites of nude mice in each group. **b** The bone mineral density (BMD) was measured on the regions of interest (ROI) of the entire defect area. **c** The bone volume/total volume (BV/TV) ratio was calculated on the ROI of the entire defect area. **d** The trabecular spacing (Tb.Sp) was measured on the ROI of the entire defect area. **e** Representative images of hematoxylin and eosin (HE) staining in the defect area. **f** Representative images of Masson’s trichrome staining in the defect area, where collagen and new bone were stained blue, and the muscle and cytoplasm were stained red. **g** Histochemistry staining of COL1A1 in the defect area (*P < 0.05, **P < 0.01, ****P < 0.000 1)

**Fig. 8:**
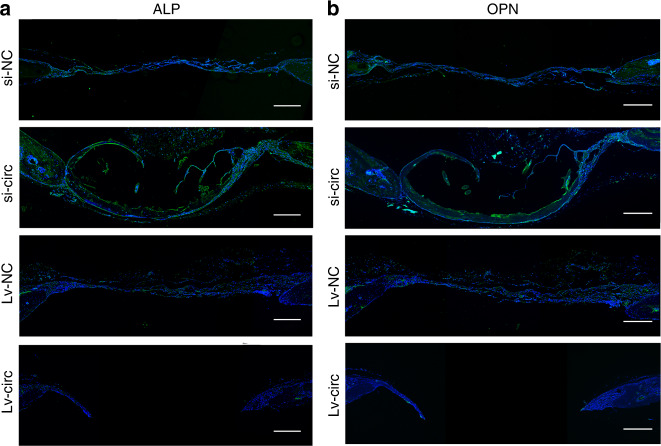
Images of immunofluorescence staining in the defect area. **a** ALP was stained in green and nuclei were stained in blue. **b** OPN was stained in green and nuclei were stained in blue. Scale bar = 250 μm

To further confirm that circ_0003204 has a similar osteogenic regulation role in vivo and in vitro, four groups of transfected hASCs were loaded on GelMA and gently implanted into the cranial defect area of nude mice (4 mm in diameter). Three-dimensional (3D) reconstructed images of the cranium were used to assess the repair of the bone defects. More newly formed bone was observed in the group of si-circ-transfected hASCs compared with si-NC, while Lv-circ-transfected hASCs decreased bone formation compared with Lv-NC (Fig. 7a). Meanwhile, the bone mineral density (BMD) and bone volume over total volume (BV/TV) were obviously promoted in circ_0003204 silencing, while reduced in circ_0003204 upregulation (Fig. 7b, c). In contrast, the value of trabecular spacing (Tb.Sp) (Fig. 7d) was reduced in circ_0003204 silencing and promoted in circ_0003204 upregulation when compared with the corresponding controls. Hematoxylin and eosin (HE) staining (Fig. 7e) for histological observations and Masson’s trichrome staining (Fig. 7f) for testing the collagen supported the microcomputed tomography (micro-CT) analysis. The results presented that new bone was formed in the defects in all groups. Compared with the control group respectively, obvious collagen deposition and more newly formed bone were observed in the group of si-circ, whereas circ_0003204 overexpression hindered the new bone growth. Histochemical staining of COL1A1, a pivotal osteogenesis-related marker involved in the deposition of collagen fiber,^19^ showed that COL1A1 expression was reduced in circ_0003204 overexpression compared with the negative control, while circ_0003204 silencing promoted COL1A1 expression in the newly formed bone area (Fig. 7g). IF staining also exhibited high expression of OPN and ALP in circ_0003204 silencing and low expression in circ_0003204 overexpression (Fig. 8a, b). These results revealed that circ_0003204 could regulate bone formation in vivo.

## Discussion

circRNAs, a new type of noncoding RNAs characterized by stable closed-loop structures, are originated from exon, intron or intergenic region.^20^ circRNAs can resist RNA external ribozyme or RNase R and are chiefly located in the cytoplasm. Increasing researches suggested circRNAs have key functions in the occurrence and development of numerous human diseases. Importantly, emerging literatures illustrated a link between circRNAs and osteogenesis. In the present research, we demonstrated that circ_0003204 regulated the osteogenic differentiation of hASCs. The in vitro and in vivo experiments confirmed that the osteogenic potential of hASCs was promoted by circ_0003204 knockdown whereas decreased by circ_0003204 overexpression.

Although substantial evidences show that circRNAs are involved in various multiple biological controls, only few reports show circRNAs regulate the osteogenic differentiation of hASCs: circFOXP1,^21^ circvgll3,^12^ circRFWD2/INO80,^8^ circPOMT1 and circMCM3AP.^4^ circ_0003204 is originated from the sixteenth and seventeenth exon of the USP36 gene, which is highly stable and is located in the cytoplasm.^22^ It was confirmed that circ_0003204 had a circular structure resistant to RNase R.^22^ Here, we confirmed circ_0003204 is a cytoplasmic circRNA in hASCs. Previous studies have detected the function of circ_0003204 in atherosclerosis.^22–27^ circ_0003204 is upregulated in human aorta endothelial cells (HAECs) under the circumstances of oxidized low-density lipoprotein and plays a key role in oxidized low-density lipoprotein-induced HAECs damage by regulating miR-370-3p/TGFβR2/phosph-SMAD3 axis,^22^ miR-330-5p/TLR4 axis,^24^ miR-942-5p/HDAC9 axis,^25^ miR-330-5p/Nod2^26^, or miR-197-3p/ROBO1 axis.^27^ In addition, circ_0003204 increases cell proliferation, migration, and invasion in patients with cervical cancer by MAPK pathway.^28^ However, no study has explored the role of circ_0003204 and specific mechanisms on osteogenesis. In this study, we illustrated its negative regulatory effects on hASC osteogenesis.

miRNAs are a type of noncoding RNAs with 20–22 nucleotides in length, which participate in the regulation of gene expression in post-transcriptional level. Previous study reported that circ_0003204 impacted biological processes through functioning as a ceRNA of miR-370.^22^ Moreover, miR-370-3p was shown to be involved in osteogenesis of human bone marrow mesenchymal stem cells (hBMSCs).^18^ Our findings also determined that the presence of miR-370-3p inhibitor inhibited the osteogenic differentiation of hASCs. Bioinformatic analysis revealed that the 3’-UTR of circ_0003204 contained miR-370-3p potential binding sites. RT-qPCR analysis showed that miR-370-3p expression was decreased after circ_0003204 overexpression, while increased after circ_0003204 down-expression. The upregulation of miR-370-3p obviously reduced luciferase activity in hsa_circ_0003204-wt transfected cells. Moreover, si-circ and miR-370-3p inhibitor co-transfection partly reversed the active function of si-circ on the osteogenic differentiation of hASCs. Furthermore, the upregulation of miR-370-3p and silencing of circ_0003204 markedly inhibited the HDAC4 mRNA expression, while the silencing of miR-370-3p and upregulation of circ_0003204 promoted HDAC4 expression. These findings revealed that the circ_0003204 sponged miR-370-3p and impacted HDAC4 expression during the osteogenic differentiation of hASCs.

HDAC4, a kind of class IIa histone deacetylase, relies on a zinc-containing folding structure for deacetylation, resulting in chromatin condensation and inhibiting gene expression.^29^ Post-translational modifications of HDAC4 such as phosphorylation can also activate its deacetylation.^30^ Several miRNAs were reported to regulate osteogenesis by targeting HDAC4. It was reported that miRNA-29a enhanced osteogenic differentiation of MSCs via targeting HDAC4 and Wnt signaling pathway.^31^ In this study, we predicted that miR-370-3p targeted the 3’-UTR of HDAC4. The results revealed the negative mRNA expression relationship between miR-370-3p and HDAC4. According to the rescue experiment, we observed that si-HDAC4 partially blocked the effects of miR-370-3p inhibitor. Likewise, we also verified that HDAC4 silencing promoted hASC osteogenesis. Previous reports have confirmed that HDAC4 acts as a corepressor on Runx2 and impacts bone formation.^32^ Our research also verified that both the mRNA and protein expressions of Runx2 were promoted in si-HDAC4. These results revealed that circ_0003204-mediated osteogenic differentiation of hASCs through the miR-370-3p/HDAC4 axis and the osteogenic function of HDAC4 on hASCs may be regulated by Runx2.

Scaffold is a three-dimensional microenvironment of bone tissue engineering, similar to the natural extracellular matrix, which can promote cell adhesion, migration, and differentiation. In the present study, we used a kind of natural hydrophilic polymer synthesized by adding methacryloyl groups into amine. It has been demonstrated that methylmethacrylate and photocrosslinking can provide tunable mechanical and chemical properties, such as the capacity to produce 3D microarchitectures.^33^ Our results indicated GelMA had good cytocompatibility for the growth of hASCs. Through the hASCs-GelMA scaffold, we confirmed that the regulation of circ_0003204 inhibited the osteogenesis of hASCs in the skull defect model.

In conclusion, our research, for the first time, explored the role of circ_0003204 in hASC osteogenesis. Our results revealed circ_0003204 could sponge miR-370-3p to regulate HDAC4 in hASCs. These findings of our research indicated that circ_0003204 might provide a novel therapeutic paradigm in bone defects repair and regeneration by hASCs.

## Materials and methods

### Cell culture and osteogenic induction

The hASCs were purchased from Cyagen (China) and were cultured in Human Adipose-derived Mesenchymal Stem Cell Basal Medium (Cyagen, China) at 37 °C and 5% CO_2_, which included 10% human adipose-derived mesenchymal stem cell-qualified fetal bovine serum and 1% penicillin–streptomycin and 1% glutamine. We used three to seven generations of hASCs for experiments. The cells were cultured in osteogenic differentiation medium at 80% confluence. The osteogenic induction medium (Cyagen, China) consisted of human adipose-derived stem cell osteogenic differentiation basal medium with 0.1 μmol·L^−1^ dexamethasone, 50 μmol·L^−1^ ascorbate, 1% β-glycerophosphate, 1% penicillin–streptomycin, and 1% glutamine.

### Quantitative real-time PCR (RT-qPCR)

TRIzol reagent (Invitrogen, USA) was applied to extract total RNA and NanoDrop ND-1000 spectrophotometer (NanoDrop Technologies, USA) was used to detect RNA concentration and quality. Subsequently, 1 000 ng of total RNA was reversely transcribed into cDNA with a PrimeScript RT reagent Kit (Takara, Japan). ABI 7500 fast real‐time PCR System (Applied Biosystems) was utilized to detect qPCR. Relative quantification was normalized and calculated by GAPDH or U6 in the formula 2^−ΔΔCt^. All Primers are listed in Supplementary Table S1.

### Separation of cytoplasmic and nuclear fractions

To verify the subcellular localization of the circRNA in the cytoplasmic or nuclear fraction of hASCs, the cell lysates were collected and separated into cytoplasmic and nuclear fractions by a nuclear and cytoplasmic extraction kit (Thermo Fisher Scientific, USA). The extracted lysates were processed for RT-qPCR and the expressions of nuclear control U6 and cytoplasmic control GAPDH were detected to determine whether it was completely separated.

### Cell transfection

The overexpression lentivirus of circRNA was subcloned into a ZsGreen reporter from Hanheng Biology (Shanghai, China) and the empty vector was acted as an NC group. The cells were transduced with lentivirus at a MOI of 100 with 2 µg/ml polybrene. Cy3‐labeled si-circ, si-HDAC4, and si-NC were designed by GenePharma Co. (Shanghai, China). Meanwhile, the mimic and the inhibitor of Cy3‐labeled hsa‐miR‐370‐3p and the relevant negative controls were generated by GenePharma Co. (Shanghai, China). Cell transfection was obtained using Lipofectamine 3000 Reagent (Invitrogen, USA) as protocol. The transfection efficacy was confirmed through both fluorescence microscopy and RT-qPCR. All synthesized sequences are shown in Supplementary Table S2.

### ALP staining and quantification

After 7-day osteogenic differentiation, samples were rinsed with PBS three times, fixed with citrate solution for 30 s, and stained by Leukocyte Alkaline Phosphatase Kit (Sigma, USA) for 15 min in the dark.

For ALP quantification, samples were washed three times with PBS and cracked with RIPA lysis buffer. Blank group, standard group, and sample group were set in the 96-well plate. Then, the samples were incubated with p-nitrophenol at 37 °C for 15 min according to the Alkaline Phosphatase Assay Kit (Beyotime). ALP activity was measured at 405 nm according to the p-nitrophenyl substrate.

### ARS staining and quantification

After 14-day osteogenic differentiation, the mineralized nodules were measured by ARS staining using 0.1% ARS staining solution (pH = 4.2, Cyagen). In brief, the cells were fixed in paraformaldehyde (4%) for 15 min. Following washing three times with distilled water, per well was applied 1 mL of alizarin red dye solution and fully reacted for 20 min.

For ARS quantification, dye solution was removed and 10% acetic acid was added for 30 min. Cells from each well were collected with a scraper. The sample was heated at 85 °C for 10 min. Then, 10% ammonium hydroxide was used to neutralize the sample to a pH of 4.1–4.5. The samples were pipetted back into a new 96‐well plate. Finally, the absorbance was calculated at 405 nm wavelength.

### Western blot

The cells were lysed by radioimmunoprecipitation (RIPA) lysis buffer and total protein was extracted. The protein contents were then detected by the BCA protein detection kit (Beyotime, China). Proteins were separated using 10% sodium dodecyl sulfate-polyacrylamide gel electrophoresis (SDS-PAGE) and then transferred onto PVDF membranes, followed by incubating overnight with the following optimal concentrations of corresponding antibodies: anti-ALP (1:500, Huabio, China), anti-OCN (1:1 000, Huabio, China), anti-RUNX2 (1:1 000, Huabio, China), anti-HDAC4 (1:1 000, Huabio, China), and anti-β-ACTIN (1:10 000, Huabio, China).

Then, the membranes were incubated with the secondary antibody (1:5 000, Huabio, China) for 2 h. Finally, the results were visualized by SuperSignal West Dura Extended Duration Substrate (Thermo Scientific, USA). All results were quantified by ImageJ software (version 6.0, National Institutes of Health), and the experiments were repeated three times.

### IF analysis of cells

After osteogenic differentiation, samples were fixed in paraformaldehyde (4%) for 15 min, permeabilized for 30 min with 0.1% Triton X-100, and then blocked using 5% bovine serum albumin (BSA) for 30 min. Subsequently, the samples were incubated with anti-RUNX2 (1:200; Huabio, China) and COL1A1 (1:200; Cell Signaling Technology, America) at 4 °C overnight and following a fluorescence-labeled secondary antibody for 2 h. Finally, the fluorescein isothiocyanate (FITC) (Solarbio, Beijing, China) stained cell body for 30 min and 4′,6-diamidino-2-phenylindole (DAPI) (Solarbio, Beijing, China) stained nucleus for 5 min. The results were recorded with a confocal microscope (Olympus FV3000, Japan).

### Dual-luciferase reporter assay

Sequences containing the potential binding sites of miR-370-3p in circ_0003204 were generated by HanBio (Shanghai, China) and inserted into PSI-CHECK2. Reporter plasmids were transduced into 293T cells. After 48 h of transfection, 100 µL passive lysis buffer was added to each well and the plate was slowly shaken for 15 min. Then, Luciferase Assay Reagent II (LAR II) (Luciferase Assay Reagent, Progema) was supplemented (100 µL per sample). After that, 20 µL cell lysis solution was added and the firefly luciferase value was recorded as control. Finally, 100 µL Stop & Glo^®^ Reagent (Luciferase Reagent, Progema) was added to each sample and Renilla luciferase value was detected.

### RIP assay

RIP was performed using the Imprint RNA Immunoprecipitation Kit (Sigma, USA). Briefly, cells were lysed in mild lysis buffer. The samples were then incubated with immunoprecipitation buffer containing protein A magnetic beads prebinding anti-Ago2 antibody (Huabio, China) or control IgG. After overnight incubation at 4 °C, the immunoprecipitated RNAs were isolated using TRIzol reagent (Invitrogen, USA) and analyzed via RT-qPCR, as described above.

### Synthesis and characterization of GelMA

GelMA (GM-90) were obtained from Engineering For Life company (Jiangsu, China). According to the instruction, 0.25% (w/v) LAP as the photoinitiator was prepared and dissolved lyophilized GelMA in a concentration of 10% (w/v). Following filtering through a 0.22-μm filter, the GelMA solution was exposed to UV light (405 nm) for 20 s. After photocrosslinking, the GelMA were frozen and the morphology was observed using a SEM (KYKY Technology Development Ltd., China). The diameter distribution of GelMA was calculated from the SEM images by ImageJ.

### The coculture of cells with scaffold and osteogenic induction

For 2D cell culture, GelMA was synthesized in the plates as mentioned and incubated in medium for 5 min. Then, the harvested cells were seeded on the GelMA and added fresh culture medium. For 3D cell culture, the cells are suspended in filtered GelMA with a density of 1 × 10^7^ cells/mL. Cell suspension was added into 96-well culture plates and exposed to UV light (405 nm) for 20 s. Then, the hASCs-GelMA were incubated in medium for 5 min and replaced with fresh culture medium. On the second day, the 3D-cultured hASCs-GelMA was transferred for osteogenic induction. After induction for 7 and 14 d, the cell scaffolds were subjected to ALP and ARS stainings, as described above.

### Biocompatibility assessment

Cell proliferation was calculated using the Cell Counting Kit-8 (CCK-8) (Dojido, Japan). Briefly, hASCs and hASCs-GelMA were seeded into 96-well plates. After culturing for 1, 2, 3, 5, or 7d, the samples were added with 20 μL CCK-8 reagents (5 g·L^−1^) and incubated for 4 h. The absorbance of samples (450 nm) was detected. The cell viability was determined by Live & Dead Viability/Cytotoxicity Assy Kit (Keygenbio, China). Five microliters of 4 mM calcein-AM was added to 10 mL PI solution. Each sample was added with 200 µL mixture and incubated for 30 min in darkness at 37 °C. Then, all samples were rinsed with PBS three times and visualized with a confocal microscope (Olympus FV3000, Japan).

SEM analysis was used to evaluate the morphology of the cells 2D-cultured on the GelMA after 3 d. Samples were fixed in glutaraldehyde (2.5%) for 1 h and dehydrated with the graded serial concentration of ethanol (30%, 50%, 70%, 80%, 90%, and 100%). After critical point dried and coating with gold, the samples were observed under a SEM (KYKY Technology Development Ltd., China).

Confocal microscopy was also used to evaluate the morphology of the cells 3D-cultured on the GelMA after 7 days. Samples were fixed in paraformaldehyde (4%) for 15 min, permeabilized for 30 min with 0.1% Triton X-100 and then blocked using 5% bovine serum albumin (BSA) for 30 min at room temperature. Then, cells were stained with FITC for 30 min and nuclei were stained with DAPI for 5 min. Finally, samples were recorded with a confocal microscope (Olympus FV3000, Japan).

### Animal surgery

All animal procedures were approved by the Animal Ethics Committee of West China Hospital of Sichuan University (Ethical number: 2021012A). In preparation for cell scaffolds, si-circ/si-NC and Lv-circ/Lv-NC were transfected into hASCs and then seeded in the GelMA to form microspheres ~4 mm in diameter. The critical-sized (4 mm) cranial defects on the nude mice were created by a dental drill after anesthesia. The scaffolds with cells were gently transplanted into the defect areas. After 8 weeks, the mice were sacrificed and the calvaria samples were collected in paraformaldehyde solution (4%) for further studies.

### Micro-CT analysis

The mice skulls were scanned by a micro-CT (Scanco, Switzerland) at a voxel size of 10 μm and a voltage of 70 kVp per 200 µA. Raw data were used for 3D reconstruction. The BMD, BV/TV, and Tb.Sp were measured.

### Histological and histomorphometric analysis

HE staining and Masson’s trichrome were used to observe morphological changes. After dehydration, decalcification and paraffin embedding, the samples were stained using Hematoxylin-Eosin Staining Kit (Solarbio, China) and Masson’s Trichrome Stain Kit (Solarbio, China). Samples were also detected by histomorphometric analysis. The specimens were permeabilized in 0.1% Triton X-100 and blocked with 5% BSA. The specimens were then incubated with anti-COL1A1 (1:200; Cell Signaling Technology, America) overnight at 4 °C.

### IF analysis of tissue sections

Antigen retrieval of tissue sections was performed at 98 °C for 30 min and blocked using 10% goat serum for 30 min at 37 °C. Then, the samples were incubated with anti-ALP (1:200; Huabio, China), anti-OPN (1:200; Huabio, China), and anti-HDAC4 (1:200; Huabio, China) overnight at 4 °C. On the next day, sections were incubated with a fluorescence-labeled secondary antibody for 1 h at 37 °C and counterstained with DAPI for 5 min. The results were observed with fluorescent microscopy (Leica, Germany).

### Statistical analysis

SPSS software (version 16.0) was used for statistical analyses in this study. All experiments were conducted in triplicate and results were reported as mean ± standard deviation (SD). Comparison among groups were achieved using the Student’s *t* test and one-way analysis of variance (ANOVA). *P* value < 0.05 was recognized as statistically significant.

## Supplementary information

Figure S1

Figure S2

Table S1

Table S2

Supplementary figure legends
