# Supplementary figures and images for: circ_0003204 regulates the osteogenic differentiation of human adipose-derived stem cells via miR-370-3p/HDAC4 axis
Source: Int J Oral Sci. 2022 Jun 21;14:30. doi: 10.1038/s41368-022-00184-2 (PMC9213414; doi:10.1038/s41368-022-00184-2)

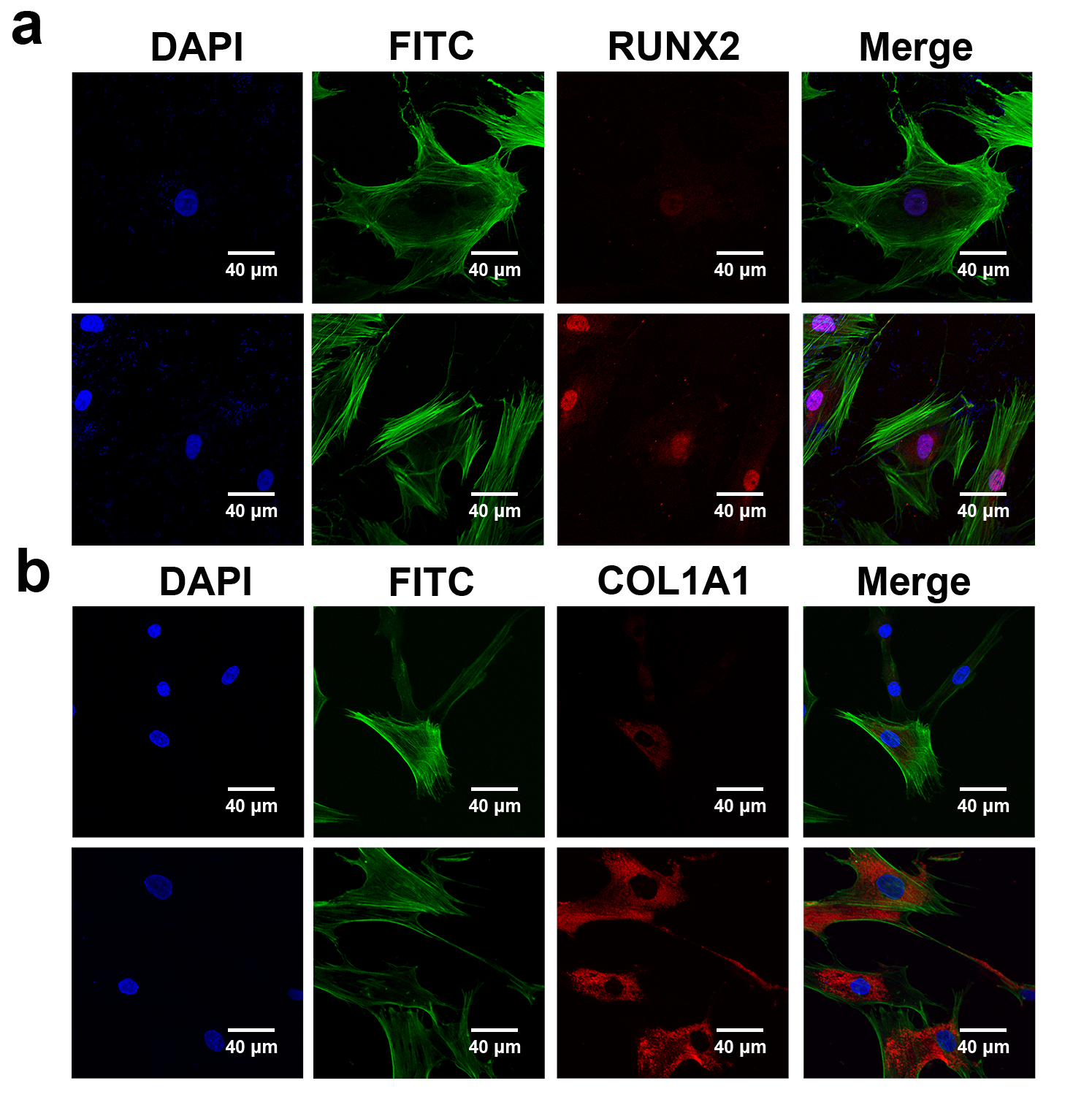

Supplement: Supplementary file 1 — Figure S1 [file 41368_2022_184_MOESM1_ESM.tif]

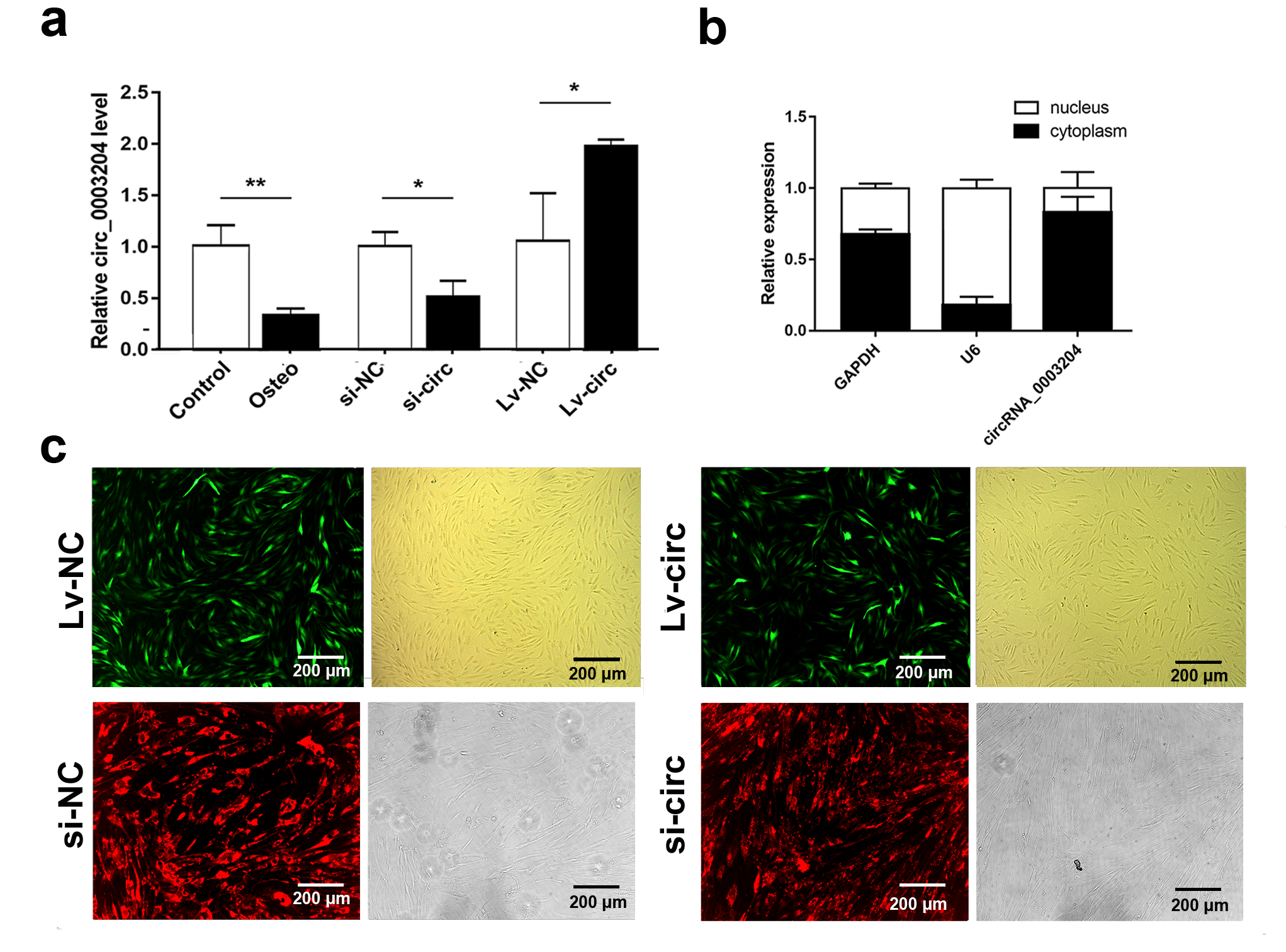

Supplement: Supplementary file 2 — Figure S2 [file 41368_2022_184_MOESM2_ESM.tif]
