# Supplementary material for: circ_0003204 regulates the osteogenic differentiation of human adipose-derived stem cells via miR-370-3p/HDAC4 axis
Source: Int J Oral Sci. 2022 Jun 21;14:30. doi: 10.1038/s41368-022-00184-2 (PMC9213414; doi:10.1038/s41368-022-00184-2)
Supplement: Supplementary file 3 — Table S1 [file 41368_2022_184_MOESM3_ESM.docx]

Table S1: Primer sequences of RT-qPCR

| Genes | Primer sequences (5’-3’) |
| --- | --- |
| **GAPDH** | Forward: CTTTGGTATCGTGGAAGGACTC |
|  | Reverse: GTAGAGGCAGGGATGATGTTCT |
| **U6** | Forward: CTCGCTTCGGCAGCACA |
|  | Reverse: AACGCTTCACGAATTTGCGT |
| **ALPL** | Forward: TTGACCTCCTCGGAAGACACTC |
|  | Reverse: CCAGGCCCATTGCCATACA |
| **RUNX2** | Forward: TGGACGAGGCAAGAGTTTCAC |
|  | Reverse: GAGGCGGTCAGAGAACAAACTAG |
| **BGLAP** | Forward: CTACCTGTATCAATGGCTGGG |
|  | Reverse: GGATTGAGCTCACACACCT |
| **HDAC4** | Forward: GCCAAAGATGACTTCCCTCTTA |
|  | Reverse: TTTCGGCCACTTTCTGCTTTAG |
| **COL1A1** | Forward: CCAGTGTGGCCCAGAAGAAC |
|  | Reverse: TGGCCGCCATACTCGAACT |
| **hsa-circ_0003204** | Forward: TACTGCCCGCTGTGTCTCCTG |
|  | Reverse: GACTGCTGCCTCTCACTTCCAAG |
| **hsa-miR-370-3p** | Forward: GCCTGCTGGGGTGGAAC |
