# Supplementary material for: circ_0003204 regulates the osteogenic differentiation of human adipose-derived stem cells via miR-370-3p/HDAC4 axis
Source: Int J Oral Sci. 2022 Jun 21;14:30. doi: 10.1038/s41368-022-00184-2 (PMC9213414; doi:10.1038/s41368-022-00184-2)
Supplement: Supplementary file 4 — Table S2 [file 41368_2022_184_MOESM4_ESM.docx]

Table S2: Synthesized sequences

| Genes | Sequences (5’-3’) |
| --- | --- |
| hsa-circ_0003204 | GACCGCAUGGGGCUGUGUCTT |
|  | GACACAGCCCCAUGCGGUCTT |
| HDAC4 | GCAGCAGCAUCAGCAGUUUTT |
|  | AAACUGCUGAUGGUGCUGCTT |
| NC | UUCUCCGAACGUGUCACGUTT |
|  | ACGUGACACGUUCGGAGAATT |
| hsa-miR-370-3p mimic | GCCUGCUGGGGUGGAACCUGGU |
| hsa-miR-370-3p inhibitor | ACCAGGUUCCACCCCAGCAGGC |
| mimics-NC | UUGUACUACACAAAAGUACUG |
| inhibitor-NC | CAGUACUUUUGUGUAGUACAA |
