# Supplementary material for: circ_0003204 regulates the osteogenic differentiation of human adipose-derived stem cells via miR-370-3p/HDAC4 axis
Source: Int J Oral Sci. 2022 Jun 21;14:30. doi: 10.1038/s41368-022-00184-2 (PMC9213414; doi:10.1038/s41368-022-00184-2)
Supplement: Supplementary file 5 — Supplementary figure legends [file 41368_2022_184_MOESM5_ESM.docx]

Figure S1 The osteogenic differentiation of hASCs. a) The osteogenic related factors COL1A1 was stained with red immunofluorescence after osteogenic induction for 7 days (scale bar = 40 μm). The 4',6-diamidino-2-phenylindole (DAPI) stained nuclei (blue) and fluorescein isothiocyanate (FITC) stained cell body (green). b) The osteogenic related factors RUNX2 was stained with red immunofluorescence after osteogenic induction for 7 days (scale bar = 40 μm). DAPI stained nuclei (blue) and FITC stained cell body (green).

Figure S2 The expression profile of circ_0003204. a) The expressions of circ_0003204 were detected via RT-qPCR. b) RT-qPCR was used to examine the expression levels of circ_0003204 in the cytoplasmic and nuclear of hASCs. GAPDH and U6 were applied as the controls and the results indicated the separation of cytoplasmic and nuclear fractions. c) The circ_0003204 lentivirus carried GFP reporter gene (green) and circ_0003204 siRNA carried Cy3 reporter (red) (scale bar = 200μm).
